# Annotations

**Published:** 1889-03-30

**Authors:** 


### ANNOTATIONS.

**Common Humanity in Lancashire.**

UNDER this heading we published a fortnight ago an annotation on the case of two men who had been removed from the Bootle Borough Hospital under what appeared to be painful and distressing circumstances. The facts, as set forth by us, were taken from the *Liverpool Evening News*. A leading article in that newspaper—which, being a local journal, ought to have been well informed—seemed to us an unexceptionable authority, and we were thence led to conclude that the bare facts at least could not be matters of dispute. We have, however, since received a communication from Dr. Beaver, house surgeon to the hospital, which throws a totally new light on the subject. The real circumstances are now stated to be briefly these: The two men referred to were admitted into the hospital, one suffering from starvation—a curable case; the other from cancer of the stomach—an incurable disease. Both men were admitted into the hospital, their condition was carefully inquired into, and they received every care and attention during the time they remained there. But the rules of the hospital do not permit of the treatment of incurable cases, except they are urgent, and the two were discharged in the ordinary way, one of them after having been four days in the hospital, the other a month. They were both in a fit condition to be discharged on the date of their removal. They were, however, destitute, and, at their own request, were sent to the relieving officer for admission to the West Derby Union. They were removed with care and kindness in a cab, and everything was done that could be done to secure their comfort and safety. How far the *Liverpool Evening News* may have been animated by purely local feelings and considerations we do not know; for our part, we had no knowledge whatever of even the names of any of the persons connected with the hospital. We expressed ourselves strongly, perhaps too strongly, seeing that we had not made ourselves acquainted with the other side of the case; but our expressions were the outcome of deep sympathy with what we supposed to be two poor and defenceless men. Our statements have been wrongly interpreted by Dr. Beaver, the Bootle house surgeon, as reflecting upon him. We sincerely regret that he should consider himself aggrieved, and are glad to have the opportunity of publishing his version of the facts. Representing, as we so largely do, hospital and medical opinion, it is no satisfaction to us, but very much the contrary, to hear of anything which seems to cast a slur upon the fair fame of hospitals or their officials. We know, too, that as a rule those officials have a high sense of duty and responsibility, and very justly resent any charge of want of kindness and consideration for their patients. Although we neither made, nor intended to make, any attack upon Dr. Beaver, he has our sincere sympathy in the circumstances in which he is placed. All men in public positions are liable to be misunderstood, and therefore to be criticised unjustly. But a plain statement of the facts, such as we here publish from Dr. Beaver, is always the best and an all-sufficient answer to such criticism.

**"Fancied Pains."**

"YOU may smile and hold such anxieties for idle fancies, like those of Don Quixote," says Heine, "but *fancied pains* do hurt all the same; and if one fancies he has drunk hemlock he may get into a consumption, and he certainly will not get fat." Imagination is a constituent part of the normal human creature, and so is the critical faculty. Just as in a state there are Conservatives and Radicals, so there are imaginative and unimaginative men and women; and just as the extreme Conservatives and extreme Radicals desire each other's complete extirpation from the face of the earth, root and branch, so do the "imaginatives" and the "criticals" fly at each others throats and gnash their furious but happily not too carnivorous teeth. Your true cynic has small patience with what he calls "hypochondriasis." The other day a little girl who was just recovering from a bronchial attack was walking in the street with her mother, carefully encased in a warm shawl. Three typical young Britons of the unimaginative class, two boys and a girl, aged respectively about eight, six, and four, were standing ready to mount their tricycles as the small beshawled invalid passed by. Their native Philistinism awoke in a moment. "Poor thing," said the eldest; "she has been ill, has she; do wrap her up well this cold day." Numbers two and three followed suit in the same tone. The incident was highly amusing, and truly characteristic of a certain class of mind. But is the man or woman who suffers from "fancied pains" to be pitied or to be blamed? "Blamed undoubtedly," says the Philistine; and if the critic happen to be at the same time a doctor or a husband, he can be the author of a very great deal of acute, but unnecessary, suffering. Imaginary pains ought to be treated seriously. They should not, indeed, be treated by means of potions and pills, but by methods of reason and sympathy. Since no outsider, not even the President of the College of Physicians, can say with certainty that the patient who complains of pain does not actually feel any, but only thinks he does, it is best at once to frankly admit the fact of pain, and then to explain that though there is pain there is no discoverable disease. Some simple remedy for giving tone to the nervous system, and a sympathetic assurance that all is well at present, but that if more serious symptoms should develop, they will be attended to, is a method of management which commends itself both to good sense and kind feeling; and generally succeeds admirably in practice. But to pooh, pooh, or sneer at or bully the unfortunate person whose imagination happens to be so active as to create symptoms which did not previously exist, is worse than stupid; it is cruel. The sympathetic doctor may be quite as honest as the cynical, and is, at any rate, a very much more agreeable man to deal with. Cynicism and scepticism are among the very cheapest and commonest wares of the intellectual market. They have their place in the mental armoury, but it is a place which is by no means always exalted.

**Mr. Gladstone's Constituents in Debate.**

PORTOBELLO is a Midlothian town whose provost and council threaten to rival in debating power the "assemblies" which annually display their rhetoric in the neighbouring city of Edinburgh, and to make the British House of Commons, in comparison, a mere college unicn. A recent subject of debate at Portobello has been one which involves the expenditure of money; and when money is to be spent your true Scotchman is all eyes and ears, as indeed he should be. It seems that a hospital for infectious diseases is needed for the town. The assembled councillors had to decide first of all whether the building should be for Portobello alone, or whether there should be a combination scheme to include Duddingston, Liberton, and some neighbouring parishes. From the point of view of economy and efficiency, and indeed from almost every other standpoint, the combination scheme is undoubtedly the best. But after a debate, which, as reported, fills nearly three columns of the local newspaper, it is impossible for a reader to understand what conclusion was arrived at, or whether there was any conclusion at all. A very considerable elevation of mental temperature was produced by the question whether the hospital was to be of brick or of stone. Now the "elect" know full well that although the native Scotchman is, of course, superior to the Englishman in every detail of his personal, public, and private life and history, yet in nothing is that superiority more conspicuously shown than in the fact that the Northerner lives in a house built of solid stone, whilst the miserable Southerner burrows and shivers in a mere habitation of brick. And yet there were not wanting what Hood calls "brazen" town councillors, and even Scotch town councillors who had the temerity to propose a "brick" hospital—aye, and to stick to it! Every loyal heart in the council was roused to a worthy indignation. One perfervid councillor and brave Horatius, McVey by name, was resolved to speak out, and to speak plainly, though he should perish in the attempt. "Those who had had," he said, "experience of a brick building said it was very unsafe for such a purpose. The brick was very porous and if they went to any brick building on a frosty day, even though the brick was lined on the inside, they would find there would likely be half-an-inch of ice inside." (O cruel reporter!) "It seemed to him that if they took patients into such an icehouse, it was just a stage between that and the cemetery." Somebody pointed out that most English people lived in brick houses; but it is well-known "thæ English" are of no account "whatever." Nevertheless, we will venture to suggest that the pleasant little Edinburgh suburb should at once provide itself with a fever hospital; that it should be according to the "combination" scheme, and that is is really a matter of no practical moment whethe it be of brick or of stone.

